# Global distribution of *Neorickettsia risticii*, the causative agent of potomac horse fever: a systematic review

**DOI:** 10.1007/s11259-026-11146-y

**Published:** 2026-03-18

**Authors:** ThankGod Emmanuel Onyiche, Tan Li Peng

**Affiliations:** 1 Departments of Veterinary Parasitology and Entomology, University of Maiduguri, P.M.B. 1069, Maiduguri, Nigeria; 2 Unit for Environmental Sciences and Management, North-West University, Potchefstroom Campus, Private Bag X6001, Potchefstroom, 2520 South Africa; 3 Faculty of Veterinary Medicine, Universiti Malaysia Kelantan, Kota Bharu, Pengkalan Chape 16100 Malaysia

**Keywords:** Aquatic insects, Bats, Digenetic trematodes, Neorickettsia risticii, Potomac Horse Fever, Soft ticks

## Abstract

Potomac Horse Fever (PHF), also known as equine neorickettsiosis (EN) or equine monocytic ehrlichiosis is an acute, potentially fatal infectious disease in horses caused by the monocytotropic rickettsia bacterium Neorickettsia risticii. This obligate intracellular bacterium is maintained throughout the life cycle of digenetic trematodes which utilize multiple intermediate and definitive hosts. Research on N. risticii is scattered across multiple continents, with most studies originating from North and South America. Therefore, a systematic review is needed to consolidate global evidence, clarify the geographic distribution and host range of this pathogen, and identify knowledge gaps to guide future surveillance, diagnosis, and control strategies. Therefore, we conducted the first systematic review of the distribution of Neorickettsia risticii in horses and invertebrates. A comprehensive search of five electronic databases was performed to retrieve studies that reported the occurrence of N. risticii. Retrieved records were screened and assessed following the PRISMA (Preferred Reporting Items for Systematic Reviews and Meta-Analyses) 2020 guidelines. Twenty-seven eligible studies published between 1990 and 2024 were included in the qualitative synthesis of this review. The reported prevalence in horses varied widely by diagnostic method: serological surveys detected N. risticii antibodies in 5.45%–26.6% of sampled populations, whereas molecular assays reported PCR positivity ranging from 0%–14.38%. Neorickettsia risticii DNA has also been detected in a variety of invertebrate and wildlife hosts, including bats, trematodes, snails, aquatic insects, and soft ticks. These findings highlight the complex ecology of N. risticii and its presence across diverse host groups, although its prevalence remains generally low. The distribution of studies was geographically skewed towards North America, revealing significant gaps in surveillance in other regions.

## Introduction

Potomac Horse Fever (PHF), also known as equine neorickettsiosis (EN) or equine monocytic ehrlichiosis, is an acute, potentially fatal disease in horses caused by *Neorickettsia* (formerly *Ehrlichia*) *risticii*, an obligate intracellular bacterium. This pathogen is an endosymbiont of digenetic trematodes (Subclass Digenea, Class Trematoda, Phylum Platyhelminthes) that parasitize snails and aquatic insects (Knowles et al. 1983; Van der Kolk et al. 1991; Madigan et al. 1997; Madigan et al. 2000). *Neorickettsia risticiii* causes horse illness and the disease is known by several names including “abdominal typhoid”, “dysentery” and “horse cholera” (Schofield 1924), acute equine diarrhea syndrome (Knowles et al. 1983), equine monocytic ehrlichiosis (Ristic et al. 1986) equine scours (*Churrido equino*) (Dutra et al. 2001) and Shasta River crud or “ditch fever” (Madigan et al. 1997).

Horses are accidental hosts, acquiring infection primarily through the ingestion of aquatic insects containing encysted, *N. risticii*-infected trematodes (metacercariae) during the summer (Madigan et al. 2000). Snails act as the first intermediate host, aquatic insects as the second, and insectivorous bats or birds serve as the definitive hosts for trematodes, facilitating the transmission of the bacterium (Chae et al. 2000; Gibson et al. 2005). Horizontal transmission from infected flukes to vertebrate tissues, including the spleen and liver of bats and birds, further suggests the potential role of these insectivorous animals as reservoirs of this parasite (Gibson et al. 2005; Vaughan et al. 2012).

The ecology of *N. risticii* is complex and DNA fragments of the bacterium have been detected in the developmental stages (egg, sporocyst, cercarial, metacercarial and adult) of digenetic trematodes (mainly of the *Acanthatrium* and *Lecithodendrium* genera) (Reubel et al. 1998; Pusterla et al. 2003; Park et al. 2003; Gibson et al. 2005; Cicuttin et al. 2022). The life cycle of these parasites is complex and involves several hosts including aquatic arthropods, aquatic snails and vertebrates. *Acanthatrium oregonense* is the adult trematode stage that *N. risticii* infects and vertical transmission of *N. risticii* from adult to egg has been reported (Gibson et al. 2005). In addition, *N. risticii* can be transmitted horizontally from infected flukes to non-parasitized tissues (particularly the spleen and liver) of bats and birds suggesting the probable role of insectivorous animals as reservoirs (Gibson et al. 2005; Pusterla et al. 2003; Vaughan et al. 2012). Gravid trematodes and eggs containing DNA of *N. risticii* have been found in the intestines of little brown bats (*Myotis lucifugus*) and big brown bats (*Eptescicus fuscus*) (Gibson et al. 2005). Adult flukes develop in the intestines of insectivorous bats and birds (Gibson et al. 2005). Furthermore, the DNA of *N. risticii* has been detected in over 13 species of both immature and adult mayflies (Ephemeroptera), caddisflies (Trichoptera), dragonflies (Odonata, Anisptera), damselflies (Odonata, Zygoptera) and stoneflies (Plecoptera) (Chae et al. 2000; Mott et al. 2002). Epidemiologically, the disease has been confirmed in North America (United States and occasionally Canada) (Heller et al. 2004; Bertin et al. 2013; Xiong et al. 2016), Brazil (Moreira et al. 2013; Paulino et al. 2020), Uruguay (Dutra et al. 2001) and Europe (Van Der Kolk et al. 1991). In Africa, the first molecular evidence of *N. risticii* in horse blood was recently using PCR (not confirmed by sequencing) in South Africa (Mlangeni et al. 2016).

Despite these research efforts, knowledge of the global distribution, host range, and transmission dynamics of *N. risticii* remains limited. A consolidated understanding of its prevalence in horses, and its presence in intermediate and definitive hosts, is crucial for guiding effective surveillance, diagnosis, and disease control strategies. Therefore, this systematic review was undertaken to compile the available literature on the occurrence of *N. risticii* in horses and other hosts worldwide, and evaluate their role in the maintenance and transmission of the pathogen.

## Materials and methods

### Study registration and search strategy

This study was conducted according to the Preferred Reporting Items for Systematic Reviews and Meta-Analyses (PRISMA) 2020 guidelines (Page et al. 2021a, b a; 2021 b). The study protocol was registered in the OSF Registries database, and is available at.

https://osf.io/d3b6q/?view_only=1abd58cd7f034bd79b9649e26435f68c (accessed on 30 November 2024). A systematic search of the literature was conducted for studies relating to the detection of *Neorickettsia risticii*, the aetiological agent of Potomac horse fever. A literature search was in four electronic databases on articles written in English language from 1st January, 1990 to October, 2024 using the keywords in combination with BORLEAN operators “OR”, “AND” to create a search string (“*Neorickettsia risticii*” OR “Potomac Horse Fever” OR “Equine neorickettsiosis”) AND (horse OR “aquatic snails” OR “aquatic insect” OR bats OR mayfly OR caddisfly) AND (serology OR molecular OR “Nested PCR”). The primary search keywords included: “Potomac Horse Fever”, “Equine neorickettsiosis”, “*Neorickettsia risticii”*, “aquatic snails”, “aquatic insect”, “bats”, “mayfly”, “caddisfly”, “Horse”. Article titles and their corresponding abstracts were scanned and relevant full-text articles were downloaded and obtained through library resources and online platforms. No attempt was made to contact the authors of the original studies for additional information and we did not retrieve unpublished articles.

### Eligibility criteria

Articles were included in this study if they met the following criteria: (i) cross-sectional studies reporting the detection or characterization of *N. risticii* in diverse hosts, including horses, aquatic insects, aquatic snails, rodents, and ticks; (ii) clearly stated the diagnostic method used; (iii) specified the country of study; (iv) were published in English between January 1990 and October 2024; (v) reported the total number of animals screened and the number of positive cases; (vi) provided the scientific name of the host animal screened; (vii) were available in full text; and (viii) included a minimum sample size of 25 animals for prevalence studies. Studies were excluded if they (i) did not focus on the detection or characterization of *N. risticii* in the relevant hosts, (ii) did not clearly report the diagnostic method, (iii) did not specify the country of study, (iv) were published in languages other than English, (v) were published before January 1990, (vi) did not report the total number of animals screened or positive cases, (vii) did not provide the scientific names of the hosts or invertebrates screened, or (viii) were experimental studies, case reports, book chapters, editorials, letters to the editor, or review articles.

### Study selection and assessment

In the first phase, titles and abstracts retrieved from the database searches were carefully screened following the removal of duplicates and irrelevant records. Full-text articles considered potentially relevant were downloaded and thoroughly evaluated against the previously defined inclusion criteria. Studies that did not meet the eligibility criteria were excluded, with reasons documented, and all studies that fulfilled the criteria were included in the qualitative synthesis.

### Data extraction and synthesis

Data were systematically extracted from each eligible study and organized using a Microsoft Excel spreadsheet. The extracted information included the author and year of the study, country, host species (including bats and rodents) with scientific names, family, genus, and species of snails, total number of snails collected, organs screened, family, genus, and species of aquatic insects, total number of insects collected, life cycle stage of the insect, diagnostic method, total sample size, number of positive cases, and estimated prevalence. Relevant themes were identified to guide the synthesis and discussion of the findings obtained from the systematic literature search.

## Results

### Study selection

**Fig. 1:**
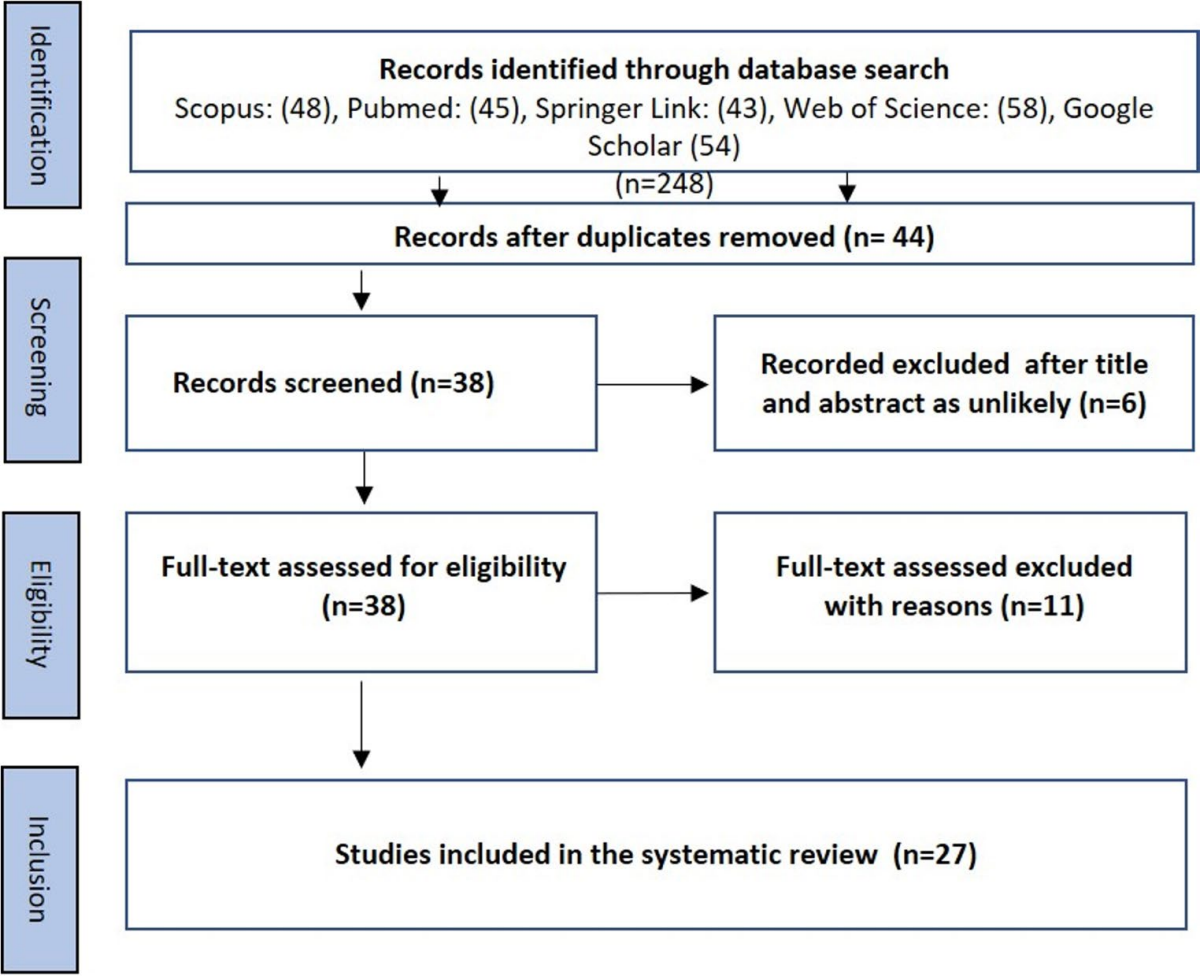
PRISMA flowchart used in the study for identification of eligible studies

A total of 248 records were retrieved from five electronic databases: Scopus (*n* = 48), PubMed (*n* = 45), SpringerLink (*n* = 43), Web of Science (*n* = 58), and Google Scholar (*n* = 54). After duplicate removal, 44 studies were included for further review. Based on the titles and abstracts, a further six studies were removed as unlikely leaving 38 studies for full-text evaluation based on the predefined eligibility criteria. Eleven articles were excluded for the following reasons: experimental design (*n* = 4), failure to detect or characterize *N. risticii* (*n* = 5), and inconsistent reporting of sample size and number of positives (*n* = 2). Ultimately, 27 studies met all inclusion criteria and were incorporated into the qualitative synthesis (Fig. 1). The 27 eligible studies were conducted across multiple continents and involved diverse hosts, including horses, bats, rodents, aquatic snails, aquatic insects, and one tick species. Diagnostic approaches varied across studies, with both molecular and serological methods being employed.

### Geographical distribution in horses

**Fig. 2:**
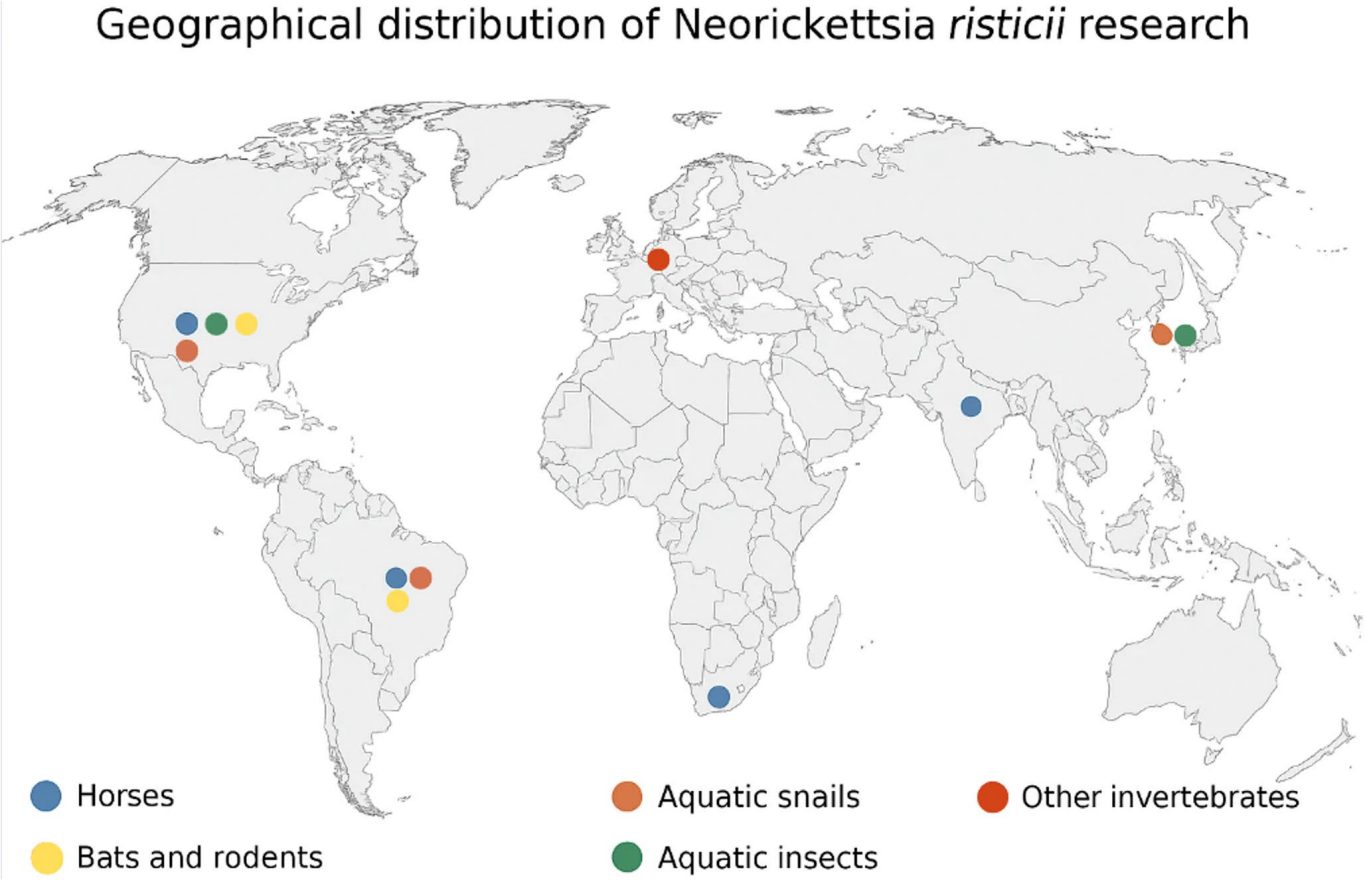
Geographical distribution of *Neorickettsia risticii* across various hosts worldwide

Of the 27 eligible studies, nine originated from only three countries, the United States, South Africa, and Brazil reflecting a geographically uneven distribution of epidemiological investigations on *N. risticii* in horses. Most studies were conducted in the United States, where Potomac horse fever is historically endemic and surveillance activity is well established. Brazil contributed a smaller but growing body of research, consistent with the increasing recognition of the disease in South America. Only one single study from Africa (South Africa) met the eligibility criteria, highlighting a major gap in surveillance across the African continent. No eligible studies were identified from Asia, Europe (apart from historical reports predating the review timeframe), Oceania, or the Middle East, indicating that global monitoring of *N. risticii* in horses remains highly concentrated in a few regions (Fig. 2).

### Host range, detection and prevalence of *N. risticii*

Nine eligible studies from the United States, South Africa, and Brazil investigated host involvement, detection, and prevalence of *N. risticii* in horses. These studies collectively represent the core evidence of *N. risticii* infection in its primary accidental host. Three studies employed PCR-based detection to identify active infections (Pusterla et al. 2000a; Mlangeni 2016; Paulino et al. 2020), whereas five studies relied on IFAT serology to determine exposure levels in equine populations (Olchowy et al. 1990; Rikihisa et al. 1990; Kiper et al. 1992; Atwill et al. 1996; Moreira et al. 2013). One study integrated both molecular and serological approaches, providing complementary insights into the infection and exposure patterns (Roier et al. 2016). Collectively, these investigations show a geographically clustered but methodologically diverse body of evidence describing the occurrence and prevalence of *N. risticii* in horses across the Americas and Africa (Table 1).

**Table 1:** Global distribution and prevalence of *Neorickettsia risticii* in horses using serological and molecular diagnostic techniques

Year of sample collection	Country	Diagnostic method	Sample size	Number of positives	Prevalence %	Author and Year of Publication
1989	United States	IFAT	318	51	16.04	Kiper et al. 1992
1986	United States	IFAT	2549	677	26.6	Olchowy et al. 1990
1986	United States	IFAT	1414	77	5.45	Rikihisa et al. 1990
	United States	IFAT	2255	164	7.30	Atwill et al. 1996
1995–1998	United States	qPCR	153	22	14.38	Pusterla et al. 2000a
2012	Brazil	IFAT	347	39	11.24	Moreira et al. 2013
NA	Brazil	IFAT qPCR,	704 704	113 0	16.05 0	Roier et al. 2016
2014–2015	South Africa	Nested-PCR	256	9	3.52	Mlangeni 2016
2012	Brazil	qPCR	187	5	2.67	Paulino et al. 2020

NA- Not available

Eight studies documented the presence of digenean trematodes and/or *N. risticii* DNA fragments in bats and, to a limited extent, in rodents, expanding the known host range beyond equines. These studies originated in Brazil (Cicuttin et al. 2013; Ikeda et al. 2021; de Mello et al. 2024), the United States (Pusterla et al. 2003; Gibson et al. 2005), Argentina (Cicuttin et al. 2017, 2022), and the Netherlands (Hornok et al. 2018). Across these investigations, PCR-based assays were applied to various organs, including the spleen, lung, liver, and blood from more than 20 bat species, reflecting the ecological importance of insectivorous bats as definitive hosts for trematodes associated with *N. risticii*. Only one study reported the detection of *N. risticii* DNA in rodent tissues, indicating limited evidence of their involvement (Table 2).

**Table 2:** Global reports on the detection and prevalence of *Neorickettsia risticii* DNA in bats and rodents

Country	Bat species	Organs tested (No. of positives/number of samples)	No. gravid trematode positive/no. trematode positive/total number	Author and Year of Publication
United States	Yuma bats (*Myotis yumanensis*)	Spleen/intestine	2/3/5	Pusterla et al. 2003
United States	Big brown bats (*Eptescicus fuscus*)	Liver/spleen (6/15) Trematode (6/12)	12/12/15	Gibson et al. 2005
	Little brown bats (*Myotis lucifugus*)	Blood (16/29)	8/8/9	
Brazil	Brazilian free-tailed bats (*Tadarida brasiliensis*)	Liver/spleen/lungs (3/30)	NA	Cicuttin et al. 2013
Argentina	Brazilian free-tailed bats (*Tadarida brasiliensis*)	Liver/spleen/lungs (5/61)	NA	Cicuttin et al. 2017
Netherland	Pond bat (*Myotis dasycneme*)	Faecal samples (4/4)	NA	Hornok et al. 2018
Brazil	*Artibeus planirostris* *Artibeus lituratus* *Platyrrhinus lineatus* *Phyllostomus discolor* *Eptesicus furinalis* *Carollia perspicillata* *Molossus molossus* *Glossophaga soricine* *Myotis nigricans* *Molossops temminckii* *Eumops perotis* *Chiroderma villosum*	Spleen/blood (10/268) Spleen/blood (11/268) Spleen/blood (11/268) Spleen/blood (9/268) Spleen/blood (4/268) Spleen/blood (3/268) Spleen/blood (2/268) Spleen/blood (2/268) Blood (1/135) Blood (1/135) Blood (1/135) Blood (1/135)	NA	Ikeda et al. 2021
Brazil	*Desmodus rotundus* *Desmodus youngii*	Spleen (24/228) Spleen (1/1)	NA	de Mello et al. 2024
Argentina	Rodent (*Oligoryzomys flavescens*)	spleen, liver and lung (1/86)	NA	Cicuttin et al. 2022

Seven studies investigated aquatic snails as the first intermediate hosts for digenean trematodes harboring *N. risticii*. Five of these studies were conducted in the United States (Barlough et al. 1998; Mott et al. 2002; Pusterla et al. 2000a, b, 2013), with additional studies from South Korea (Park et al. 2003) and Brazil (Costa et al. 2016). Using nested PCR or qPCR, researchers detected *N. risticii* DNA across several snail genera, including *Juga*, *Elimia*, *Semisulcospira*, *Radix*, and *Planorbella*. The reported prevalence varies widely among species and geographical locations, underscoring the ecological diversity of snail hosts involved in the transmission cycle (Table 3).

**Table 3:** Global overview of Infection rates for trematodes and Snails with *Neorickettsia risticii* DNA

Country	Year of sampling	Detection technique	Total number of snails collected	Family, Genus or Species of Snail	Family, Genus or Species of metacercaria	Number of snails infected with cercariae	Number positive with *N*. risticii DNA	Author and Year of Publication
United States	1996	Nested PCR	180	Lymnaeidae	NA	NA	0	Barlough et al. 1998
				Physidae	NA	NA	0	
				Planorbidae	NA	NA	0	
				Pleuroceridae	NA	NA	2	
United States	2000	Nested PCR	42	*Elimia virginica*	NA	NA	5	*Mott et al. 2002
United States	1999	Nested PCR	720	*Juga Yrekaensis* (small snail) (*n* = 360)	NA	NA	5	Pusterla et al. 2000a
				*Juga Yrekaensis* (Large snail) (*n* = 360)	NA	NA	24	
United States	1995–1998	Nested PCR	234	*Juga* spp.	NA	NA	25	Pusterla et al. 2000b
South Korea	2001	PCR	3219	*Semisulcospira libertine* (*n* = 2778)	Schistosomatidae	69	11	Park et al. 2003
					Microphallidae	28	5	
					*Echinostoma cinetorchis*	36	0	
					*Metagonimus yokogawaii*	4	0	
					Trichobilharzia	4	0	
					*E. hortense*	10	0	
					Furcocercus	20	0	
					Xiphidiocercaria	139	34	
					Unknown	52	10	
			368	*Radix auricularia coreana* (*n* = 368)	*E. cinetorichis*	16	6	
					*E. hortense*	36	2	
					*Fasciola* spp.	13	1	
					Schistosomatidae	1	0	
			73	*S. gottschei* (*n* = 73)	*E. cinetorchis*	5	0	
United States	2010–2011	qPCR	568	*Juga* spp. (*n* = 218)	NA	NA	1	Pusterla et al. 2013
				*Planorbella subcrenata* (*n* = 140)	NA	NA	3	
				*Physella virgata* (*n* = 210)	NA	NA	0	
Brazil	2013	PCR	410	*Melanoides tuberculate* (*n* = 294)	*Megalourous* spp. (*n* = 3) *Pleurolophocercous* spp. (*n* = 2)	5	0	Costa et al. 2016
				*Pomacea* spp. (*n* = 45)	NA	0	0	
				*Drepanotrema anatinum* (*n* = 15)	NA	0	0	
				*Biomphalaria tenagophila* (*n* = 27)	*Furcocercous* spp.	7	0	
				*Biomphalaria straminea* (*n* = 1)	NA	0	0	
				*Physa acuta* (*n* = 23)	NA	0	0	
				Hydobriidae (*n* = 5)	NA	1	0	

NA: Not available

* pooled samples and the cercariae and sporocyst were screened

Three studies have examined the role of aquatic insects as second intermediate hosts carrying trematode metacercariae infected with *N. risticii*. Two studies from the United States (Mott et al. 2002; Chae et al. 2000) and one from South Korea (Park et al. 2003) used nested PCR to detect *N. risticii* DNA in multiple insect species. Positive detections were reported across different life stages, including larvae, nymphs, and adults of mayflies and caddisflies. It is likely, however speculative that these insects may facilitate accidental ingestion by horses (Table 4). One additional study expanded the known invertebrate host range by reporting *N. risticii* DNA in *Ornithodoros hasei* larvae, a soft tick species, using PCR (Ikeda et al. 2021). Although this finding represents an isolated report, it highlights the potential for broader invertebrate involvement in the transmission of *N. risticii*.

**Table 4:** Global distribution of trematode metacercariae in aquatic insect carrying *Neorickettsia risticii* DNA

Country, year of sampling	Detection technique	Insect (order)	Stage of insect	Family, Genus or Species of Flies	Number of flies collected	Number of metacercaria	Number of positive to *N*. risticii DNA	Author and Year of Publication
South Korea, 2001	Nested PCR	NA	Adult	*Sympetrum darwinianum*	211	1103	0	Park et al. 2003
		NA	Adult	*Symptrum eroticum*	6	0	0	
		NA	Adult	*Symptrum parvulum*	23	51	0	
		NA	Adult	*Calopteryx japonica*	70	131	0	
United States, 2000	Nested PCR	Mayfly (Ephemeroptera)	Larva	*Leucrocuta Minerva*	10*	NA	2	Mott et al. 2002
		Caddisfly (Trichoptera)	Larva+	*Cheumatopsyche campyla*; *Hydropsyche hageni*	8*	NA	3	
United States	Nested PCR	Caddisfly (Trichoptera)	Larva	Limnephilidae (*Dicosmoecus* spp.)	144	NA	65	Chae et al. 2000
				Limnephilidae (*Oncosmoecus* spp.)	4	NA	0	
				Hydropsychidae (*Hydropsyche* spp.)	2	NA	1	
			Adult	Sericostomatidae	8	NA	4	
				Leptoceridae	49	NA	20	
		Mayfly (Ephemeroptera)	Nymph	Heptageniida (*Heptagenia* spp.)	44	NA	13	
				Tricorythidae (*Tricorythodes* spp.)	11	NA	1	
				Ephemerellidae (*Ephemeralla* spp.)	5	NA	0	
				Baetidae	32	NA	0	
		Damselfly (Odonata, Zygoptera)	Nymph	Coenagrionidae (*Argia* spp.)	26	NA	11	
				Coenagrionidae (*Telebasis salva Hagen*)	11	NA	1	
				Calopterygidae (*Hetaerina americana Fabricius*)	10	NA	0	
			Adult	Coenagrionidae (*Argia* spp.)	20	NA	0	
				Coenagrionidae (*Enallagma* spp.)	10	NA	0	
				Calopterygidae (*Calopteryx aequabilis say*)	11	NA	1	
				Calopterygidae (*Hetaerina vulnerata Hagen*)	16	NA	2	
				Calopterygidae (*Hataerina americana*)	11	NA	1	
		Dragonfly (Odonata, Anisoptera)	Adult	Libellulidae (*Erythrodiplax*)	10	NA	1	
		Stonefly (Plecoptera)	Nymph	Perlodidae (*Skwala* spp.)	30	NA	24	

NA: Not available

* pooled sample

## Discussion

Since the first detection of the disease in horses along the Potomac River in 1979, the epidemiology, mode of transmission and reservoir host of *N. risticii* are still unfolding (Madigan et al. 2000). Of all *Neorickettsia* species, *N. risticii* is the most geographically widespread species causing Potomac horse fever in horses (Greiman et al. 2015). The reported prevalence in horses varies with diagnostic methods: serological surveys range from 5.45% to 26.6%, while molecular detection ranges from 0% to 14.38%, influenced by season, sample size, vector availability, and horse health. Infection with *N. risticii* can cause acute enterocolitis, abortion in pregnant mares, and laminitis in horses. Early diagnosis allows prompt treatment and reduces the impact of the disease (Cicuttin et al. 2013; Dutra et al. 2001; Coimbra et al. 2005).

### Pathogenesis, cross species impact and transmission of *Neorickettsia risticii*

**Fig. 3:**
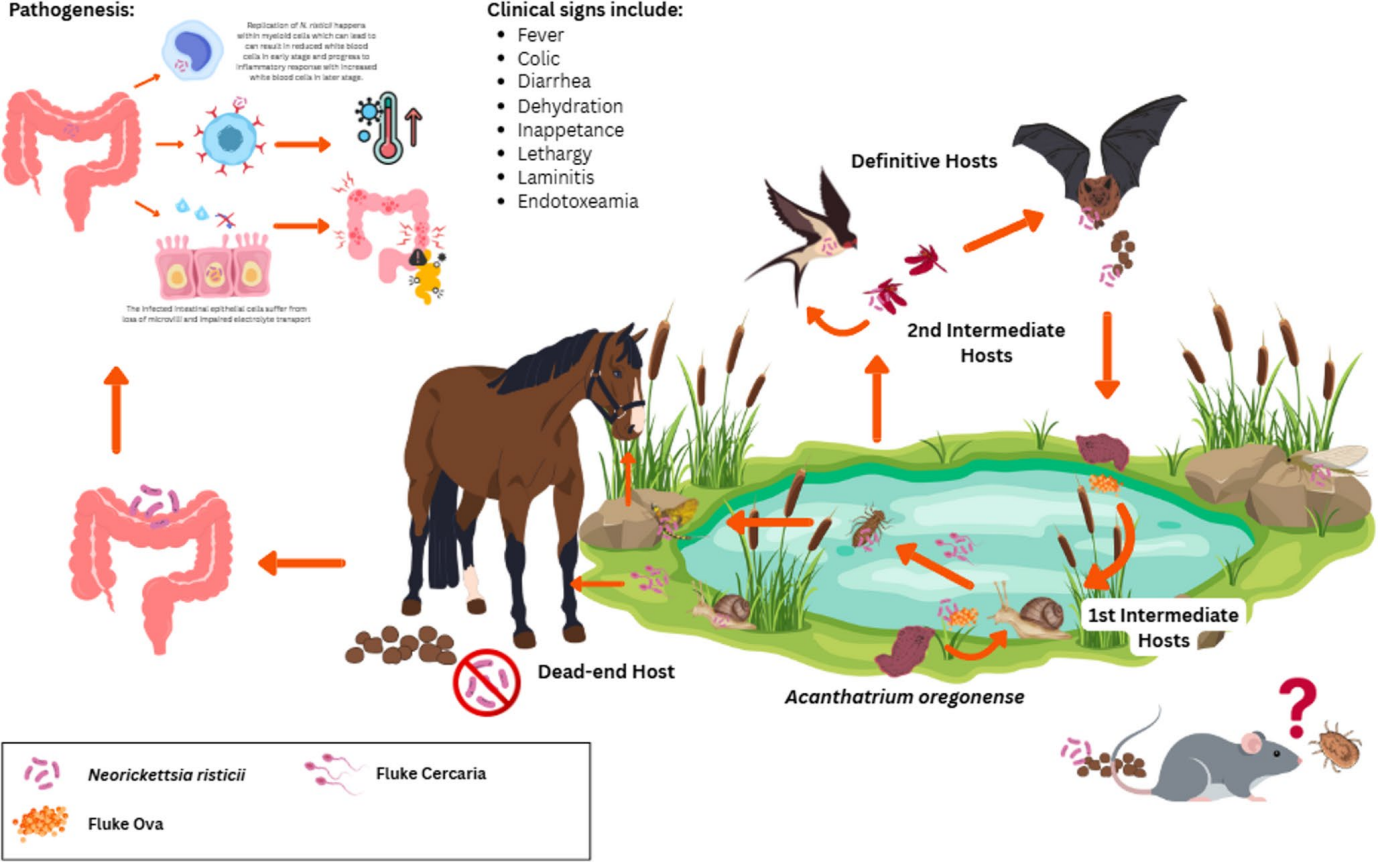
Pathogenesis and transmission cycle of *Neorickettsia ristii* in horses and across various hosts

Following ingestion, *N. risticii* is released into the gastrointestinal lumen, multiplies, and invades colon epithelial cells (Rikihisa et al. 1985, 1992; Gibson and Rikihisa 2008). The bacterium primarily infects cells of the mononuclear system, including monocytes, macrophages, mast cells, and intestinal epithelial cells causing an acute systemic disease, that can be potentially fatal (Rikihisa et al. 1992; Gibson and Rikihisa 2008). Clinically, PHF is characterized by colitis, often accompanied by fever, anorexia, depression, dehydration, diarrhea, laminitis, and occasionally, abortion (Rikihisa 2004; Bertin et al. 2013; Long et al. 1995a, b; Coffman et al. 2008). Hematological abnormalities such as neutropenia and elevated hematocrit, as well as biochemical alterations including hyperbilirubinemia, hypochloremia, hyperglycemia, hyponatremia, and hypoalbuminemia, have also been reported (Bertin et al. 2013). A schematic representation of these events is presented in Fig. 3. Furthermore, *N. risticii* or its DNA has been detected in a wide range of hosts beyond clinically affected horses. Horses remain the primary host in which infection leads to recognized disease, supported by PCR- and serology-based investigations in the United States, South Africa, and Brazil. However, the presence of this organism in bats, rodents, aquatic snails, aquatic insects, and certain invertebrates illustrates a far more complex ecological system (Fig. 3). Multiple studies have reported DNA fragments of *N. risticii* in bats, often across numerous organs such as the spleen, lung, liver, and blood (Gibson et al. 2005; Cicuttin et al. 2017; Hornok et al. 2018; Ikeda et al. 2021). Rodents were evaluated less frequently, with only one study reporting positive results (Cicuttin et al. 2022). These observations raise important questions regarding whether wildlife acts as incidental hosts, passive carriers of trematodes, or part of a larger transmission network of trematodes. Although this organism has been detected across numerous taxa, clinical disease has primarily been documented in horses (Greiman et al. 2015). The wide ecological range of *N. risticii* and its trematode vectors suggest that horses may be at risk in more areas than previously recognized, especially in regions where digenean-infected snails and aquatic insects are abundant. The detection of this organism in new geographical regions indicates the potential for future emergence or unnoticed transmission cycles, reinforcing the need for surveillance in equine populations.

### Usefulness of biological sample type and diagnostic approach for confirmation of infection

Detection of *N. risticii* relies on serological and molecular approaches (Ristic et al. 1986; Biswas et al. 1991). The indirect fluorescent antibody (IFA) test, developed in 1986, remains widely used (Ristic et al. 1986); however, it cannot distinguish between past and current infections or vaccine-induced antibodies. Antibody kinetics are complex, with rapid increases occurring even before clinical signs appear (Palmer 1993; Baird and Arroyo 2013). Molecular methods, particularly PCR and nested-PCR, are commonly used to detect *N. risticii* DNA in blood or feces, with nested-PCR being more sensitive for peripheral blood (Bertin et al. 2013; Pusterla et al. 2013; Mott et al. 1997; Paulino et al. 2020; Fortin-Trahan et al. 2023). Combining serological and molecular techniques provides a more robust diagnostic approach for confirming clinical infections.

### Role of bats and rodents as reservoirs of *Neorickettsia risticii*

Aside from horses, *N. risticii* DNA has been detected in multiple organs and feces of various bat species and swallows across Europe, North and South America (Gibson et al. 2005; Cicuttin et al. 2017; Hornok et al. 2018; Ikeda et al. 2021). In the USA, infected bats include *Myotis yumanensis*, *Eptesicus fuscus*, and *M. lucifugus* (Pusterla et al. 2003; Gibson et al. 2005), whereas in Brazil and Argentina, the Brazilian free-tailed bat (*Tadarida brasiliensis*) is primarily implicated (Cicuttin et al. 2013, 2017). In Europe, only one report has involved the pond bat (*M. dasycneme*) (Hornok et al. 2018). Detection of the bacterium in bat organs suggests that bats may act as reservoirs, potentially via adult trematodes that transmit *N. risticii* through their life cycle (Cicuttin et al. 2013). Prevalence varies among bat species, sample types, and methods (Cicuttin et al. 2013).

Phylogenetic analyses of the *p51* and 16S rRNA genes sequences showed that horse-derived isolates clustered with bat-associated fluke isolates, indicating the circulation of genetically related strains across hosts (Cicuttin et al. 2017; Hornok et al. 2018). Recently, *N. risticii* DNA was detected in the spleen of a hematophagous bat (*Desmodus sp*.) in Brazil and in soft ticks (Ikeda et al. 2021), highlighting its broader circulation (de Mello et al. 2024). Vampire bats may acquire pathogens via blood feeding, although genome-level studies are needed to confirm their identity. Experimental studies have not supported the role of ticks or other hematophagous arthropods in transmission over the past three decades (Levine et al. 1992; Hahn et al. 1990). Thus, a recent report on the detection of *N. risticii* DNA in soft ticks (*Ornithodorus hasei*) may reflect contamination rather than true vector competence (Ikeda et al. 2021).

Other than bats, the only rodent known to carry *N. risticii* is *Oligoryzomys flavescens*, which is distributed across Uruguay, Brazil, and Argentina. This rodent primarily feeds on seeds, with molluscs and insects seasonally supplementing its diet (Polop et al. 2003; Barquez et al. 2006; Navone et al. 2009). Detection of *N. risticii* DNA may reflect bacterial translocation or intestinal contamination (Giannelli et al. 2014). Phylogenetic analysis indicates that the strain in this rodent is related to, but distinct from, classical *N. risticii* (Cicuttin et al. 2022).

### Role of aquatic insects as infection source in the transmission of *Neorickettsia risticii*

Trematodes use snails as their first intermediate hosts and aquatic insects such as mayflies and caddisflies as their second intermediate hosts. Insects can transmit *N. risticii* by serving as prey for reservoir hosts, sustaining infected trematode populations, and transporting the pathogen to terrestrial habitats after metamorphosis (Chae et al. 2000). Adult insects can retain metacercariae and directly infect horses and other predators. The seasonal emergence of flying aquatic insects coincides with PHF outbreaks in spring and early summer (Usinger 1968). Prevalence in insects varies with species, life stage, and environmental factors, and most studies have been conducted in the USA, with limited evidence elsewhere (Chae et al. 2000; Mott et al. 2002; Park et al. 2003). Further studies are needed to establish the role of insects in the epidemiology of Potomac horse fever.

### Role of snails in the transmission of *Neorickettsia risticii*

Snails the first intermediate hosts are crucial for the transmission of *N. risticii*. Sporocysts, cercariae, and metacercariae from the snails of the genera *Elimia*, *Juga*, *Radix*, *Planorbella*, and *Semisulcospira* harbor the DNA of this bacterium’s (Barlough et al. 1998; Pusterla et al. 2000a, b; Mott et al. 2002; Park et al. 2003). Horses are exposed to infected cercariae through ingestion from water, encysted cercariae on vegetation, or via infected aquatic insects (Barlough et al. 1998). In South Korea, two species of snails; *Semisulcospira libertine* and *Radix auricularia coreana* have been reported with molecular evidence of *N. risticii* (Park et al. 2003). *Semisulcospira libertina* is an important intermediate host of digenitic trematodes and *N. risticii* was detected by PCR in two species of echinostomatoid trematodes that infect snails (Park et al. 2003). The prevalence in snails ranges from 0% to 20% and is influenced by season, region, and snail and trematode development (Pusterla et al. 2000a, 2013). Observations of caddisfly larvae associated with *Juga spp*. support the role of aquatic insects in seasonal PHF transmission (Barlough et al. 1998).

### Diagnostic techniques employed in Investigation

There are three main diagnostic categories: PCR-based detection, serological assays (primarily IFAT), and combined PCR and serology (Biswas et al. 1991). Horses were the only hosts for which all methods were applied, revealing how different diagnostic tools influence the perceived prevalence. PCR-based studies have identified fragments of the pathogen or infections, whereas IFAT has detected exposure but not necessarily active infection (Palmer 1993; Baird and Arroyo 2013). The presence of both positive PCR and serological results in some regions highlights the variability in diagnostic sensitivity and local transmission intensity. In wildlife (bats and rodents) and invertebrates, PCR is the predominant technique, reflecting the need to target molecular fragments in hosts where serology is impractical (Bertin et al. 2013; Pusterla et al. 2013; Mlangeni 2016; Paulino et al. 2020). The use of nested PCR and qPCR in snails and aquatic insects enhanced sensitivity, enabling the detection of trematode-associated DNA fragments even when the prevalence appeared low.

## Conclusion and limitations

In this review, we highlighted the global distribution and prevalence of *N. risticii* in two vertebrate hosts, horses and bats, and in invertebrate hosts such as aquatic insects and snails. The data indicate that North and South America are the primary endemic regions, while PCR detection have suggests that the pathogen may circulate in South Africa (Africa) and the Netherlands (Europe). Screening for this pathogen has employed both serological and molecular techniques in equids and other vectors worldwide. Therefore, increasing awareness of the global status of equine neorickettsiosis is essential to inform authorities and stakeholders of the need for improved preventive and control measures. The interpretation of these findings is limited by several factors in the literature. The sampling was uneven across countries, with the United States contributing to the majority of studies. The absence of studies in various regions may likely reflects surveillance gaps and publication bias and not a true absence of the pathogen. Also, articles published in other languages except English were excluded as stated in the eligibility criteria. This could have led to the exclusion of important studies with interesting findings further limiting our knowledge on the epidemiology of the disease. The absence of extensive epidemiological studies limited the study to a systematic review only without meta-analysis. Host representation was also skewed, with extensive research on horses but comparatively few studies on wildlife (bats) and invertebrates. Variability in diagnostic methods further complicates comparisons across regions and host species, as many studies rely solely on PCR and provide no information on active infection or antibody response. Additionally, most wildlife studies have examined DNA fragments in isolated tissues without assessing live infections or trematode burdens. Future research should focus on establishing standardized diagnostic protocols across regions and host species to facilitate more robust comparisons. Expanded surveillance of wildlife, particularly bats, rodents, and understudied invertebrates, is necessary to clarify their roles in the transmission of this pathogen. Investigations linking trematode identity, snail species, and insect hosts to *N. risticii* DNA detection are essential for understanding vector competence. Finally, longitudinal studies in regions with only sporadic reports, such as South Africa, the Netherlands, and Argentina, are needed to determine whether *N. risticii* is established or represents an occasional introduction.

## Data Availability

No datasets were generated or analysed during the current study.
